# Design, Analysis, and 3D Printing of a Patient-Specific Polyetheretherketone Implant for the Reconstruction of Zygomatic Deformities

**DOI:** 10.3390/polym15040886

**Published:** 2023-02-10

**Authors:** Khaja Moiduddin, Syed Hammad Mian, Usama Umer, Hisham Alkhalefah, Faraz Ahmed, Faraz Hussain Hashmi

**Affiliations:** 1 Advanced Manufacturing Institute, King Saud University, Riyadh 11421, Saudi Arabia; 2 Department of Mechanical Engineering, College of Engineering, King Saud University, Riyadh 11421, Saudi Arabia

**Keywords:** zygoma, cranium, implant reconstruction, PEEK, 3D printing, fitting accuracy analysis, finite element analysis

## Abstract

The reconstruction of craniomaxillofacial deformities, especially zygomatic bone repair, can be exigent due to the complex anatomical structure and the sensitivity of the crucial organs involved. The need to reconstruct the zygomatic bone in the most precise way is of crucial importance for enhancing the patient outcomes and health care-related quality of life (HRQL). Autogenous bone grafts, despite being the gold standard, do not match bone forms, have limited donor sites and bone volume, and can induce substantial surgical site morbidity, which may lead to adverse outcomes. The goal of this study is to provide an integrated approach that includes various processes, from patient scanning to implant manufacture, for the restoration of zygomatic bone abnormalities utilizing Polyetheretherketone (PEEK) material, while retaining adequate aesthetic and facial symmetry. This study takes an integrated approach, including computer-aided implant design using the mirror reconstruction technique, investigating the biomechanical behavior of the implant under loading conditions, and carrying out a fitting accuracy analysis of the PEEK implant fabricated using state-of-the-art additive manufacturing technology. The findings of the biomechanical analysis results reveal the largest stress of approximately 0.89 MPa, which is relatively low in contrast to the material’s yield strength and tensile strength. A high degree of sturdiness in the implant design is provided by the maximum value of strain and deformation, which is also relatively low at roughly 2.2 × 10^−4^ and 14 µm. This emphasizes the implant’s capability for load resistance and safety under heavy loading. The 3D-printed PEEK implant observed a maximum deviation of 0.4810 mm in the outside direction, suggesting that the aesthetic result or the fitting precision is adequate. The 3D-printed PEEK implant has the potential to supplant the zygoma bone in cases of severe zygomatic reconstructive deformities, while improving the fit, stability, and strength of the implant.

## 1. Introduction

The zygomatic bone is a diamond-shaped irregular bone that defines the anterior and lateral surface of the face. It plays a crucial role in masticatory functioning and maintains aesthetic beauty [1]. Zygomatic bone reconstruction with an implant is one of the most difficult procedures in implant dentistry due to its free-form anatomical structure. Although autogenous bone grafts are the gold standard, they do not match bone shapes and have a significant risk of bone resorption and surgical site morbidity, which can result in less-than-appealing results [2]. Standard-sized implants that are mass-produced have minimal success since they do not precisely match the curves of the bone. A little mismatch between the shapes of the implant and the bone most often lead to implant instability, surgical revisions, and implant failure. The primary factor in the surgical long-term solution of zygomatic reconstruction is accurate restoration of the zygomatic implant [3].

The two-dimensional (2D) radiographic images from Computer Tomography (CT) or Magnetic Resonance Imaging (MRI) scan data can now be transformed into three-dimensional (3D) printable files to generate unique anatomical structures due to advances in imaging technology and computer-aided medical modeling software. When compared to physically adjusting or bending the implant, the precision of the customized implants made with computer-aided design/computer-aided manufacturing (CAD/CAM) technology can help with proper placement of the prosthetic implant with streamlined operations. Custom-designed implants produce very accurate implant fitting, permit equally-distributed mechanical stress over the anatomical region, and prevent the axial and torsional instabilities linked to aseptic loosening and failure, as seen in commercial implants. Patient-specific implants have a number of advantages over conventional implants, such as quicker recovery times after surgery, a lower risk of surgical revisions, a shorter post-operative rehabilitation period, fewer surgical complications, lower treatment costs, and the absence of the patient’s psychological discomfort.

Additive manufacturing (AM) technologies, also known as 3D printing, allows direct digital manufacturing of the patient-specific implants of complex anatomical structures with high precision and productivity. Three-dimensional printing technology is also significantly faster and less expensive when compared to conventional manufacturing techniques such as milling, casting, and machining [4]. Three-dimensional printing technologies such as selective laser sintering (SLS), fused filament modeling (FFM), selective laser melting (SLM) and electron beam melting (EBM) can use biocompatible material such as titanium, polyetheretherketone (PEEK), Ti6Al4V, stainless steel, and Cobalt chromium to facilitate the fabrication of implants [5]. 

PEEK is a member of the polyaryl-ether-ketone family and was synthesized for the first time in 1978 [6]. It is an advanced high polymer material that has been employed as a reliable alternative to other alloplastic biomaterials due to its excellent chemical resistance, fatigue resistance, lightweight, high yield strength, and biocompatibility [7]. PEEK provides a number of benefits: it is bone-friendly and its elastic modulus is closer to the bone compared to metallic implants such as titanium and zirconium [8,9]. The use of PEEK in cranio-maxillofacial reconstruction has been reported in several studies in the literature with favorable outcomes [10,11]. Several researchers have reported the use of customized PEEK implants with satisfactory aesthetic and clinical results when compared to metallic implants [12,13]. Metal implants result in streak artifacts during CT scan procedures. The long-term presence of metals inside the human body results in hypersensitive reaction and can also initiate osteolysis [14]. Metallic implants, due to their high elastic modulus, induce a stress-shielding effect along the surrounding bone tissue, leading to prosthesis loosening and implant failure. PEEK is lighter than other bio-metals and thus has the advantage of less stress shielding effect on the bone and can move along with bone freely. With a tensile strength of 80 MPa and an elastic modulus of nearly 3–4 GPa, the mechanical properties of PEEK are closer to that of the human cortical bone, which has a tensile strength of 104–121 MPa and an elastic modulus of roughly 14 GPa [15]. PEEK has been used as medical implants in the reconstruction of cranial, mandibular, orthopedic and dental surgeries [16,17,18,19]. Subtractive manufacturing methods such as computer numerical control (CNC) and injection molding were used to manufacture the PEEK material, but it had many challenges such as material hardening with slightly lower temperatures and a significant amount of wastage [20,21]. Three-dimensional printing of PEEK using FFM allowed the fabrication of complex and free form anatomical structures with simple, cost-effective and hassle-free steps [22]. 

Although fabrication of PEEK has been investigated by several researchers, its application as a medical implant using 3D printing is still challenging due to its physical properties [23,24]. In this study, FUNMAT HT Enhanced (Intamsys, China), a high-temperature fused filament fabrication (FFF) device, is used to fabricate the PEEK zygomatic implant. The methodology adopted in this study involves patient-specific zygomatic implant design, biomechanical analysis of stress–strain distribution, FFF method of PEEK fabrication, and implant fitting accuracy. 

## 2. Materials and Methods

**Figure 1:**
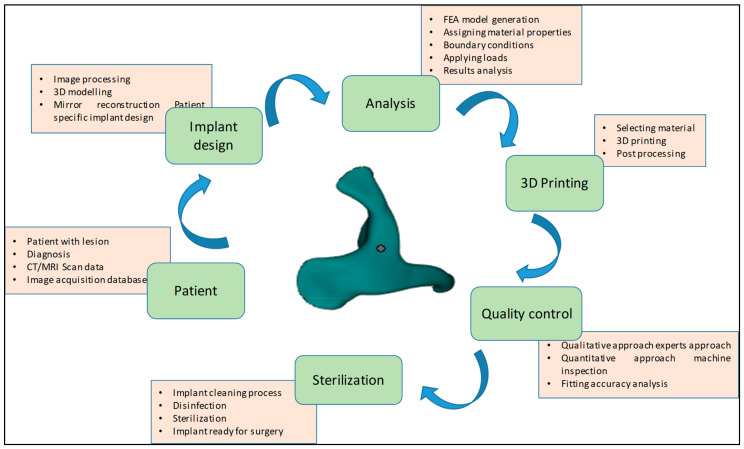
Methodology involved in the fabrication of patient-specific PEEK zygomatic implant for maxillofacial deformities.

The methodology presented for the reconstruction of patient-specific zygomatic implant consist of six stages as shown in Figure 1. The first phase consists of collecting the patient data, which includes the patient CT/MRI (computer tomography/Magnetic resonance imaging) scan, and storing the required information in a sharing database. The second phase consists of patient-specific implant design using medical modeling software. The third phase consists of biomechanical analysis of the generate finite element (FE) model to study the stress and strain distribution of the PEEK implant. The fourth phase consists of 3D printing of the zygomatic implant using PEEK material through FFF technology. The fifth phase consists of a quality check of the implant and its fitting accuracy and, finally, in the last phase, the produced PEEK implant is sterilized for surgical intervention. 

### 2.1. Image Data Collection and 3D Model Generation

**Figure 2:**
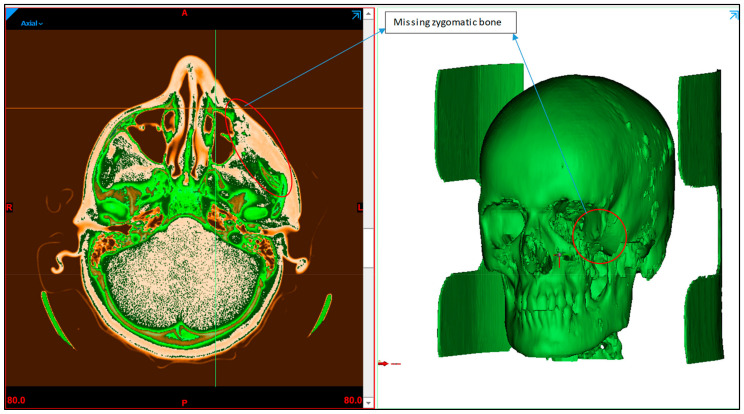
Radiographic image displaying the zygoma bone deformation in the left maxillofacial region.

A patient with midfacial bony defects is considered in this study. The pathological conditions that were found to occur in the zygomatic bones include infections, systemic diseases, metabolic diseases, bone diseases, neoplasms, and trauma [25]. Together with the facial bone, the zygomatic bone forms a nearly quadrangular structure and is a key component of mastication. Additionally, the zygomatic bone aids in the movement of the jaw and safeguards the facial nerves, organs and blood vessels. The patient transverse, sagittal and coronal data of the skull are obtained using standard CT scanning procedures. The scan data enable the observation of the detailed internal anatomical structure in a set of 2D images, which are stored in a DICOM (Digital imaging and communication field of medicine) format. DICOM is the international standard for storing medical images data. A sequence of 2D images was converted into a digital 3D model by importing the DICOM file into the medical modeling program Mimics 18.0 (Materialise, Leuven, Belgium). As shown in Figure 2, the radiographic 3D model generation clearly displays bone deformity on the left cheek.

### 2.2. Implant Design

**Figure 3:**
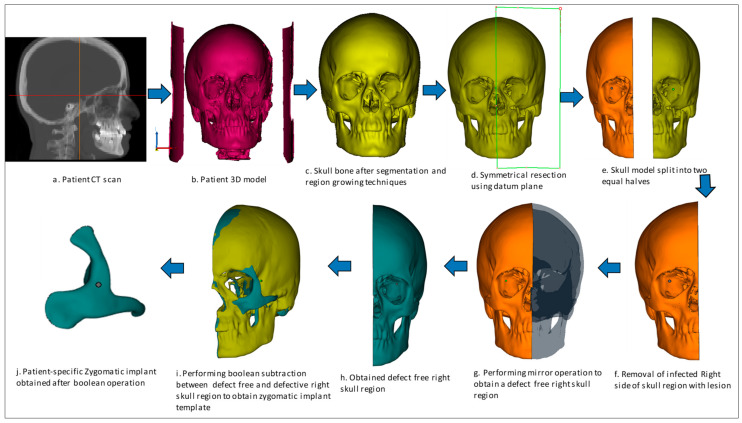
Design process flow for the generation of Patient-specific zygomatic implant from CT scan data.

The implant design procedure is based on the CAD approach. The obtained 3D model (Figure 3b) from the CT scan (Figure 3a) was subjected to segmentation and region-growing techniques using Mimics to segregate the unwanted regions and obtain the desired region of interest on the skull model (Figure 3c). The resection operation was performed based on the center datum plane (Figure 3d) to split the skull model into two equal halves (Figure 3e). The symmetrical plane was defined based on the patient CT scan data of the coronal, axial and 3D model. The two end corners of the 3D model were taken as the landmarks and based on the landmarks, the cutting plane was drawn. The cutting plane acts as a template for the midsagittal plane for resecting the skull model into two halves. The slightest deviation can result in a significant error, hence repeated attempts were made to ensure the closest symmetrical model with minimum deviation was obtained. Virtual 3D evaluation was performed to ensure the obtained implant model was the closest to the actual part with minimum deviation. The infected bone loss of the left half was removed (Figure 3f) and the mirror imaging technique was performed (Figure 3g) to mirror the healthy right side or defect free region (Figure 3h) over the contralateral region. Next, a Boolean subtraction operation (Figure 3i) was performed between the left region of the skull with the defect and defect-free region in order to generate the implant template (Figure 3j). After boolean subtraction, a few simple cleaning and smoothing operations were performed on the implant model to remove bad edges and contours. As per the doctor’s suggestion, specific screw positions were selected for implant stability.

### 2.3. Finite Element Analysis

FE analysis has proven to be a useful tool to predict the biomechanical performance of the designed implant and to predict the success of the implant under clinical conditions [26]. A complicated structure is broken down into smaller components using FE analysis, and each of these smaller components is mathematically described as nodes and elements. The smaller the elements, the greater the value of accuracy, but in return, it takes more time to analyze the problem. FE analysis helps engineers and designers improve the design model and prevent future accidents by finding the weak spots and areas of instability [27].

FE analysis is carried out using ABAQUS/CAE (Version 6.14, Dassault Systemes, Veliez-Villacublai, France) to assess the biomechanical stability and safety of the 3D printed PEEK implants. The generated FE model of the implanted skull is put through loading and boundary conditions. The PEEK zygomatic implant’s behavior under actual operating settings is simulated using the FE analysis. Three components make up the computational FE model taken into consideration in this study: the skull, the PEEK zygomatic implant, and the implant fastening screws. The characteristics of the materials assigned to the FE model are listed in Table 1. 

In the initial step, solid Initial Graphics Exchange Specification (IGES) models for both the Zygoma implant and the skull are created using the triangular faceted Standard Tessellation Language (STL) files. All preprocessing for the FE models was carried out using ABQUS/CAE.

**Figure 4:**
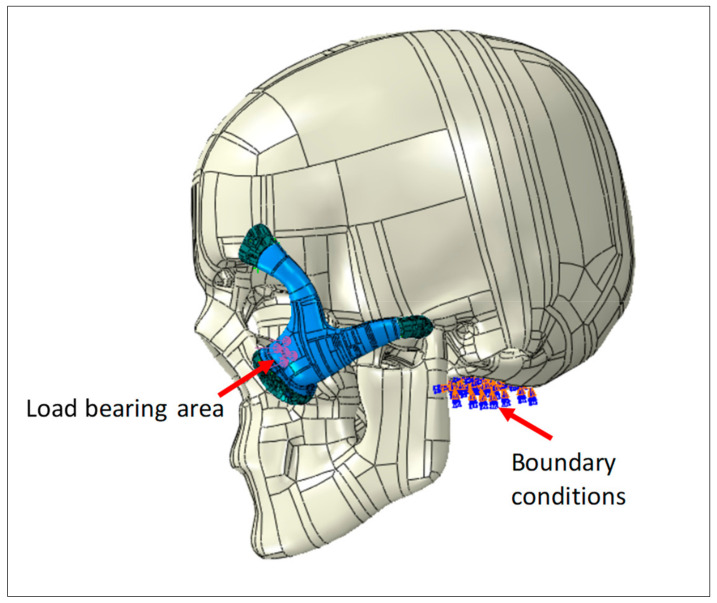
Zygoma implant and the skull loading and boundary conditions.

The necessary boundary conditions and the applied load is described in Figure 4. Full encastre boundary condition was used around the neck area. The load bearing area is around 500 mm^2^, selected at the central portion of the implant with a total force of 50 Newton. 

Point-based connections between the two surfaces can be modeled using a Mesh independent fastener provided by ABAQUS/STANDARD. This is the most convenient and robust method to model connections such as spot welds. The joints between the implant and the skull were simulated using these fasteners by selecting the portion of the surfaces to be joined and the spot weld positions. In total, 12 fasteners were utilized and distributed across the joining surfaces, as depicted in Figure 4. 

**Figure 5:**
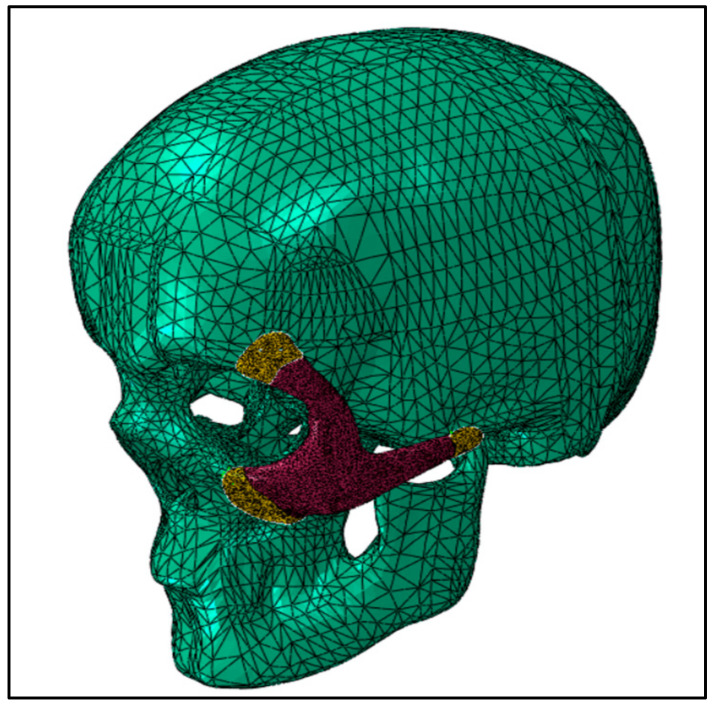
FE mesh of the skull and zygoma implant.

The continuum quadratic tetrahedron elements in Abaqus/Standard (C3D10) with 10 nodes were used for the mesh assembly in the static general procedure, as shown in Figure 5. Mesh sensitivity analyses were performed, and in order to reduce computation time, an ideal element size was chosen, yielding around 150K elements for the model assembly. Frictionless contact was assumed between the skull and the implant, and the general contact algorithm for 3D surfaces was selected. 

### 2.4. Fabrication Process

**Figure 6:**
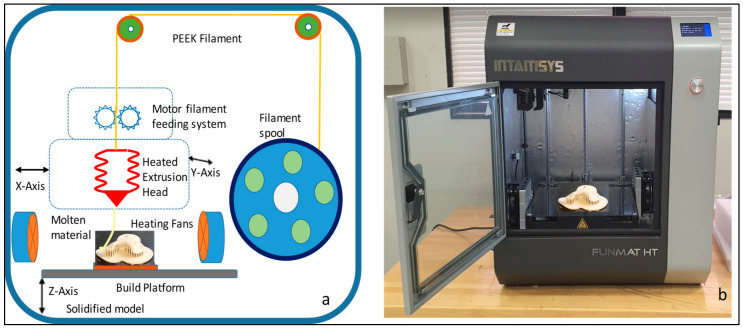
(**a**) Schematic diagram of the Intamsys printer process and (**b**) Intamsys funmat HT printer used for the fabrication of PEEK zygoma implant.

Intamsys FUNMAT HT 3D printer (Intamsys Technology Co., Ltd., Shanghai, China) is used in this study, which works on the principle of FFF, whereby the filament (PEEK) is fed from a spool through a heated extruder head and deposited on a built platform as shown in Figure 6. The INTAMSYS^®^ PEEK material spool with a filament diameter of 1.75 mm is used in this study for the fabrication of the zygomatic implant [30]. The extruder or print head moves in two dimensions X and Y as per the CAD model using computer control and prints the defined shape. After completion of each shape, the print head is lowered vertically in the Z direction to begin a new layer of material.

The zygomatic implant STL file is imported into the Intamsuite 3.6.2, a slicing CAD software of INTAMSYS. The STL file is sliced into horizontal layers for the print. Raft build plate adhesion assistant is used beneath the implant for a stronger adhesion base. The Zygomatic implant model is printed under high accuracy with a layer thickness of 0.1 mm and 100% infill with a gyroid layer pattern. The printer performance details are provided in Table 2. The nozzle follows a raster pattern, and after each print the working bed is lowered, and a new layer is extruded on top of the existing layer. Support structures are incorporated as necessary depending upon the build model. The printing takes place under a closed chamber with a bed temperature of 160 °C, chamber temperature of 120 °C and print temperature of 420 °C. To avoid warping issues during the build process, special adhesion glue was applied in a crosshatch pattern on the heated build platform to secure the print model. Additionally, the build plate adhesion type raft was used to add an extra thick grid around the implant to increase the surface area on the bed.

**Figure 7:**
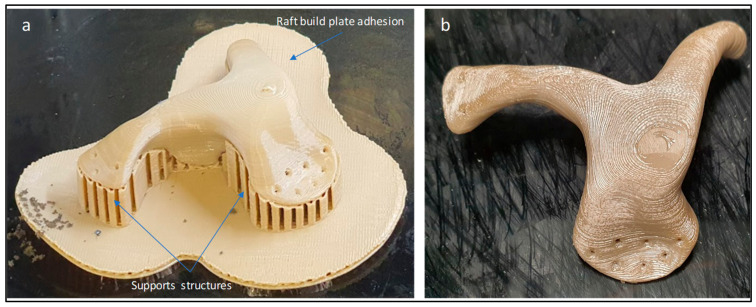
(**a**) Zygomatic PEEK implant with supports structures and (**b**) implant after removal of supports.

After completion of build, the build platform is allowed to cool down before removing the printed build model. In post-processing, the zygomatic implant support structures are removed manually using cutting pliers and tweezers. Figure 7 illustrates the zygomatic PEEK implant with supports and after the removal of the supports. The time taken to complete the PEEK print is around 2 h, 45 min, and the printing cost is approximately 40 USD.

### 2.5. Implant Fitting Analysis

A pleasant and precise implant fit is necessary for acceptable and feasible zygomatic restoration. When a face feature, such as the cheek, has a malformation, the accuracy of the implant fitting becomes very important. Implant fitting analysis is therefore necessary to enhance implant placement and the overall attractiveness of the facial anatomy.

**Figure 8:**
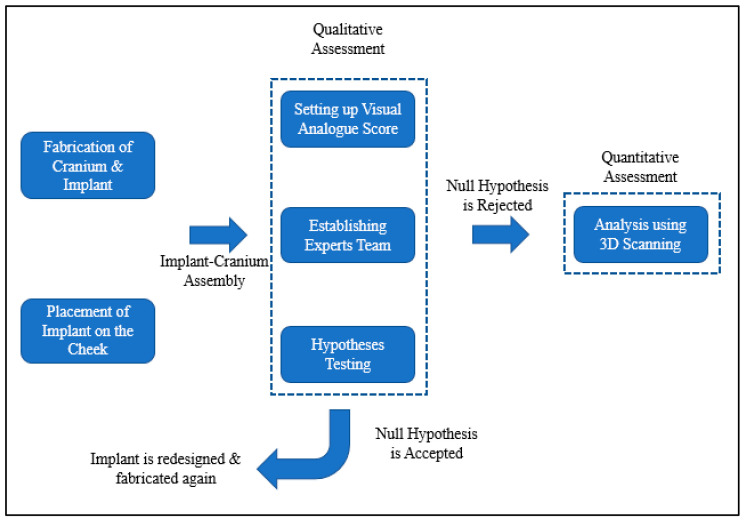
Framework adopted to evaluate the implant’s fitting accuracy.

**Figure 9:**
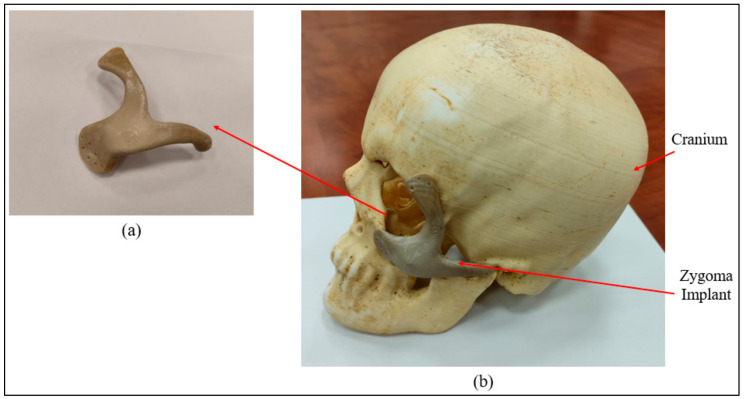
(**a**) Intamsys-produced PEEK Zygoma implant (**b**) Cranium and Zygoma implant assembly.

Figure 8 provides information on the procedure used to assess implant fitting. Acrylonitrile butadiene styrene (ABS), a widely utilized thermoplastic polymer, was employed to build the cranium structure. As shown in Figure 9a,b, the PEEK implant was fitted on a polymer cranium framework for fitting assessment. A visual analogue score (VAS) of 1 to 5 (1, poor; 2, average; 3, satisfactory; 4, good; 5, exceptional) was employed to measure the aesthetic results of the implant’s placement on the cranium and its alignment [31]. The implanted cranium was presented to five medical professionals and five research professionals from the department of surgery. A total of three PEEK implant models were produced and given to the specialists for inspection. Each analyst was asked to autonomously review each replication by looking at the assembly in the context of cranium consistency, alignment, and aesthetic appeal. Each model was subsequently given a visual score ranging from 1 to 5 from each judge. Consequently, the median aesthetic score was estimated, which is the average of each expert score. The null hypothesis and the alternative hypothesis are the two hypotheses that demonstrated in this study for a statistical explanation [32,33]. The alternative hypothesis is that the mean aesthetic score is higher than 3 (Ha: µ >3), while the null hypothesis states that the median aesthetic score is less than or equal to 3 (H0: µ ≤ 3). The one-sample *t*-test in Minitab was applied to the hypothesis testing (Minitab 17, USA). The implant was then evaluated using the 3D scanner on the portable coordinate measuring machine (CMM), in the case that the null hypothesis was not accepted. If the null hypothesis was accepted, the implant was revised and produced once again using 3D printing.

To determine divergence from the reference mirrored geometry, a quantitative assessment of the PEEK Zygoma implant’s fitting accuracy that is produced by FFF using PEEK (Intamsys) was also conducted. The mirror image of the cranium was generated by reflecting the right cheek, which is in good health, onto the left cheek to make up for the left side’s deficiency. The mirrored cranium used in this study is presumed to be perfect and it differs from the patient’s cranium insignificantly when the patient was healthy.

**Figure 10:**
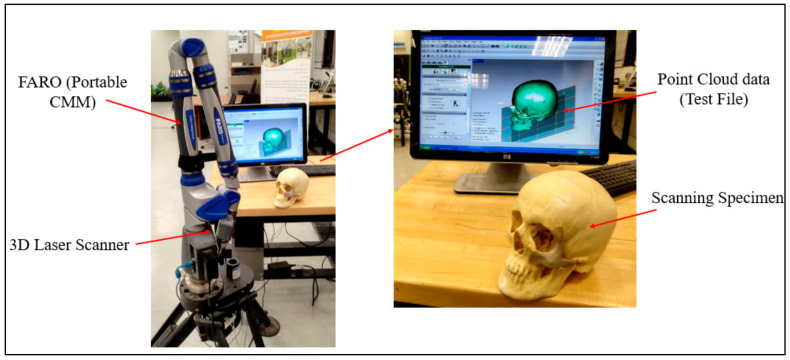
Data capture by deploying FARO arm (portable CMM).

As shown in Figure 10, the FARO arm laser scanner (Faro, Lake Mary, FL, USA) was deployed to scan the FFF-produced ABS cranium and the PEEK implant assembly for inspection. In this investigation, specialized Geomagics Control software (3D Systems Inc., Cary, NC, USA) with a 3D comparison application was chosen [34,35]. The 3D comparison method characterizes the distance from the analyzing surface to the equivalent reference surface [36]. The 3D comparison is one of the most precise and dependable methods available for examining the surface variations between the test (analyzing) and the reference CAD models [37]. It is regarded amongst the most accurate and cost-effective techniques for estimating errors and illustrating surface irregularities [38].

The outer surface of the implanted cranium (STL) was imported into Geomagics Control as a test model and matched with the reference CAD model (mirrored). The outside (outer) surface of the implant was considered since the tailored implants were designed in conformity with their exterior (outer) surface. The Geomagics software quantifies the shortest distance between the test and the reference models to display the result. Three primary steps make up the 3D comparison approach.

**Figure 11:**
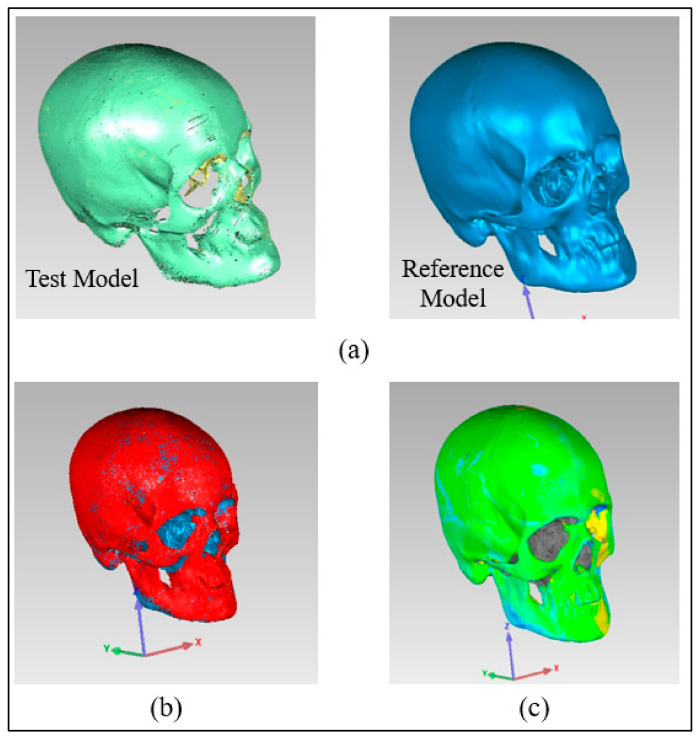
Pictorial representation of three primary steps (**a**) Allocation of entities as reference and test (**b**) Alignment (**c**) 3D Comparison.

- Assignment of entities as test and reference. The data gathered for the fabricated sample (Zygoma implant fitted on the cranium) were imported into the software as the test model, while the mirrored cranium model was set as the reference (Figure 11a).
- Alignment. The reference model and the implanted test model were aligned so that they were placed in the same coordinate system. Using the best-fit alignment, the implant-cranium reconstruction model was positioned relative to the mirrored cranium model (Figure 11b). The best-fit alignment of the test and reference surfaces ensures that all components are placed in the same coordinate system [39]. It suggests that the test surface should overlay its idealized counterpart as close as feasible. The least-square method aligns the two surfaces by projecting one of the two surfaces so that the sum of the squared distances between them at matching locations is the shortest. It adaptably decreases the distance among each point on the test surface and its reference.
- 3D Comparison. It effectively represents the difference between the test and reference models (Figure 11c).

### 2.6. Implant Sterilization

PEEK Implants must be sterilized prior to implantation and should also withstand years of aqueous environmental exposure at body temperature. PEEK offers a standard sterilization process including steam, gamma radiation and Ethylene Oxide (EtO) sterilization [40]. Studies reveal that neither steam nor gamma radiation sterilization process have any adverse effect on the mechanical properties of PEEK material [41]. The printed Zygomatic implant endures a series of cleaning processes before sterilization to remove any unwanted organic and inorganic containment molecules from the surface of the implant. The steam sterilized zygomatic implant is stored in a temperature-controlled vacuum sealed wrap prior to surgery. 

## 3. Results and Discussion

**Figure 12:**
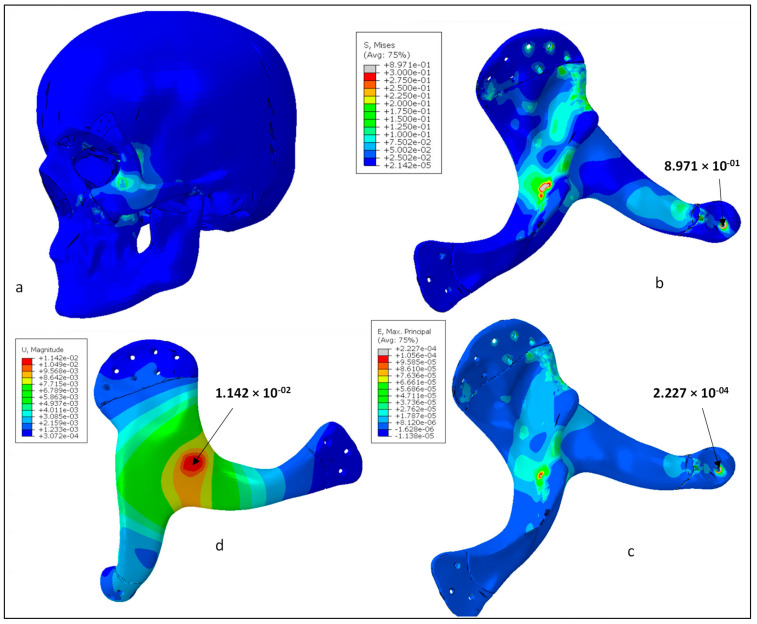
(**a**) Von Mises stress distributions in the whole model, (**b**) Von Mises stress distributions for the zygoma implant (MPa), (**c**) Maximum principal strain for the zygoma implant, (**d**) Total displacement contour for the zygoma implant (mm).

Figure 12a shows the Von mises stress contours for the skull and zygoma implant. As shown in the figure, contact areas and the loaded region were found with high stresses. In contrast, other areas of the implant had negligible stresses. Von Mises stress contour only for the implant is shown in Figure 12b. The highest stress occurred at one of the joint areas at around 0.89 MPa, which is obviously very low in comparison to the material yield strength and tensile strength (90/100 MPa). This ensures the high rigidity of the implant design and absence of any fracture or failure for the prescribed load and boundary conditions. Similarly, Figure 12c shows the maximum principal strain contour for the zygoma implant. As highlighted, the strain values were quite low and maximum strain was around 2.2 × 10^−4^. It can be noticed that both stress and strain contours are similar and maximum values are coincident. Finally, the deformation contour for the zygoma can be examined in Figure 12d. As expected, maximum deformation occurred at the loading position. However, the magnitude was very low, i.e., around 14 µm, and reduced to zero near the fastening positions.

The mean aesthetic outcome during the implant fitting procedure was better-looking, scoring 3.40 out of 5 (*n* = 10). A *p* value less than 0.05 indicated that the result was statistically significant. The one-sample *t*-test was used for hypothesis testing. The null hypothesis was rejected because the *p* value (=0.006) was less than the significance level (α) of 0.05, and the average aesthetic score was greater than 3, indicating the expert’s satisfaction and confidence. This highlights how well the implant fits the cranium and produces a pleasing cosmetic appearance.

**Figure 13:**
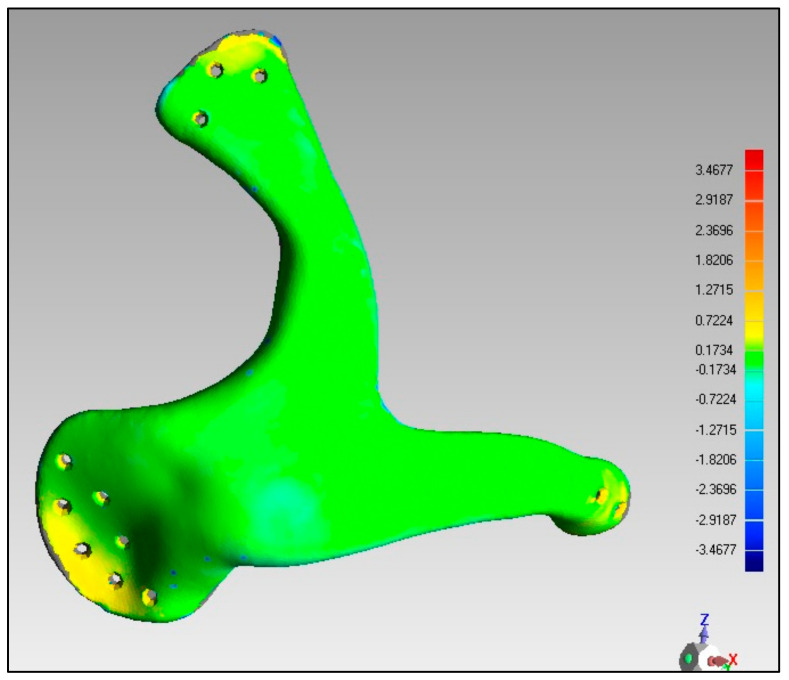
Deviation analysis results of the fabricated implant.

The quality of the manufactured Zygoma implant was also quantitatively estimated by applying 3D comparison analysis in Geomagics Control. As depicted in Figure 10, the custom designed Zygoma implant was positioned on the skull and scanned to collect the test results. Three-dimensional scanning was accomplished by utilizing the laser scanner mounted on the Faro Platinum arm (FARO, Florida, USA). The fabricated zygoma implant after support removal was also inspected in comparison to its designed equivalent before the zygoma implant-skull was examined. This was carried out to quantify the 3D printing error. Figure 13 illustrates the 0.12 mm variation between the fabricated and the implant geometry.

**Figure 14:**
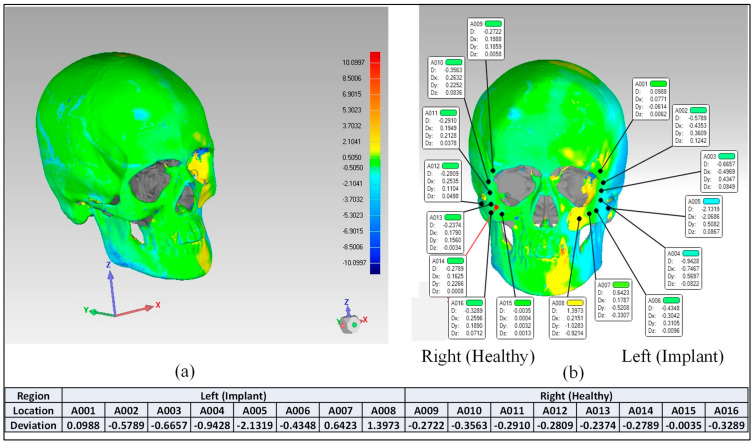
Fitting accuracy (**a**) Entire cranium (**b**) Region of interest (Zygoma implant).

After the deviation analysis of the fabricated implant, the implant was fitted to the skull model to examine the overall aesthetic outcomes of the reconstructed skull. Figure 14a graphically illustrates the outcomes of the 3D deviation study for the zygoma implant-skull. A good fit between the implant and the hosting bone is crucial for implant stability and long-term success, and a mean gap of 0.5 mm will establish acceptable implant-bone contact [42]. When comparing the reconstructed cranium to the mirrored cranium, the overall divergence in the outer direction is 0.4810 mm. Similarly, the overall difference between the region of interest as illustrated in Figure 14a,b Zygoma implant on the left side—and the right healthy side is only 0.2729 mm, which is fairly low given the intricate anatomy of the area. The fitting accuracy of the fabricated PEEK implant is 0.48 mm, which is very similar to the mean deviation of 0.41 mm 3D-printed bio-ceramic zygomatic implant, as reported by Lee et al. [43]. Although some deviation values were greater than 0.5 mm (see Figure 13), the mean of all the individual deviation values should not exceed 0.5 mm. Numerous factors could have led to some individual deviation values above the 0.5 mm limit. These may include a slight inaccuracy made when determining the symmetry plane, a 3D printing error or the other flaw made while removing supports, an operator inconsistency committed when fixing the implant to the skull, a scanning error, etc.

In light of above two analyses, the rebuilt cranium is suitable and provides sufficient aesthetics and attractiveness. Very few studies that explore the permissible tolerance range for medical implants have been published in the literature. This investigation is one of the few initiatives to gauge the implant’s fitting precision. To make sure the conclusions are reliable, two methodologies depending on quantitative and qualitative concepts are applied. An expert panel typically assesses the implant quality in the outset, as is customary. Once the specialists are satisfied with the design and quality, the implants are produced and quantitatively validated using 3D scanning. The degree of tolerance for medical implants that is actually acceptable is the one that makes the patient content and enables them to lead fulfilling lives. The process used to evaluate implant accuracy in this study will help patients obtain the intended cosmetic outcomes, while lowering psychological stress, the need for surgical revisions, and financial costs. 

The mechanical properties of PEEK achieve 99.9 MPa in tensile strength and 3738 MPa in Young’s Modulus, which is closer to that of human bone, thus reducing the risk of bone resorption and stress shielding effect on the surrounding bone. The surgical time in the reconstruction of zygomatic bone using customized PEEK implants should take less time as there is no need to harvest bone from elsewhere in the body and the patient discharged time, and the hospitalization period will also be reduced due to no donor site morbidity.

## 4. Conclusions

Zygoma bone replacement with an implant is one of the most challenging procedures in implant dentistry due to the anatomical structure’s free-form nature. The implant design, material choice, and fabrication are the three main factors in cranial reconstruction because there are so many different materials and fabrication techniques available. A compact, simple-to-fit, sturdy implant with the optimum cosmetic and functional outcomes should be produced by the material and production approach. In this study, a customized zygomatic implant was designed based on the CT scan and 3D printed from PEEK material. FE analysis was used to assess the implants biomechanical stability. Implant fitting analysis was also carried out to enhance implant placement and the inherent elegance of the facial anatomy. 

The biomechanical study revealed that the PEEK implant experienced maximum stress of approximately 0.89 MPa, which is extremely low in relation to the material’s yield strength and tensile strength (90/100 MPa). The strain values were also quite low, with a maximum strain of about 2.2 × 10^−4^. Maximum deformation was also less, at about 14 µm. This guarantees that the design of the implant is highly robust, stable and emphasizes the implant’s capacity for load resistance and safety under heavy loading. The implant’s fitting analysis was carried out using two evaluation methods (qualitative and quantitative), which proved that the implant’s variance from the cranium is not more than 0.4810 mm, which is fairly low as per the intricate anatomy of the area. The FFF-fabricated bespoke PEEK zygoma implant successfully recovers the facial expression, attractiveness, and bone shapes. Because PEEK material offers improved load bearing capabilities and a reasonable fitting accuracy, it can be used as an alternative to titanium implants. This study demonstrates the feasibility and promising value of the patient-specific 3D-printed PEEK implant for cranio-maxillofacial bone surgeries. The authors have made every effort to locate the symmetry plane in the structure at the ideal location, but there is still scope to design algorithms that can do so effectively and efficiently. Further studies involving biomechanical and surgical intervention of PEEK implants is also necessary to precisely investigate its strength in clinical applications and to become an integral part of large mainstream hospitals and clinics. In the future, in vitro and in vivo studies of the porous PEEK implants would demonstrate its osseointegrative properties, cell proliferation, and its subsequent cell adhesion around the tissues.

## Figures and Tables

**Table 1:** Material characteristics utilized in the finite element model [28,29].

Materials	Youngs Modulus (MPa)	Poisson’s Ratio	Yield Strength (MPa)
Cortical bone	13,700	0.3	122
PEEK implant	3738	0.4	99.9
Titanium screws	120,000	0.3	930

**Table 2:** Intamsys printer setting for the fabrication of PEEK zygoma implant.

Printer Setting for PEEK Material	
Nozzle diameter (mm)	0.4
Layer Thickness (mm)	0.1
Nozzle Temperature (°C)	430
Heated-Bed Temperature (°C)	160
Chamber Temperature (°C)	120
Infill Pattern	Grid
Support Pattern	Zig Zag
Build Plate adhesion	Raft
Print Speed mm/Sec	50
Slicing software	IntamSuite 3.6.2

## Data Availability

The data presented in this study are available in the article.
